# Wood Coloration and Decay Capabilities of Mycoparasite *Scytalidium ganodermophthorum*

**DOI:** 10.3390/jof9070738

**Published:** 2023-07-11

**Authors:** Ray C. Van Court, Leon Rogers, Seri C. Robinson, Gerald Presley

**Affiliations:** Department of Wood Science, Oregon State University, Corvallis, OR 97331, USA; ray.vancourt@oregonstate.edu (R.C.V.C.); leon.rogers@oregonstate.edu (L.R.); seri.robinson@oregonstate.edu (S.C.R.)

**Keywords:** fungal pigment, spalting, soft rot, wood decay, natural colorant

## Abstract

*Scytalidium ganodermophthorum* (telomorph: *Xylogone ganodermopthora*) Kang, Sigler, Lee & Yun is a destructive fungal pathogen that produces a yellow pigment that is used in sustainable product development. Similar pigmenting ascomycetes cause soft rot in woody substrates, however, the decay capabilities of *S. ganodermophthorum* have not been assessed or related to pigment production. A wood block decay test showed highly variable production of the expected bright yellow pigment and a secondary darker pigment when tested against multiple wood species and nutrient conditions. Microscopic examination showed cell wall erosion typical of type-2 soft rot in wood, although enzymatic analysis did not show detectible levels of endocellulase. Chitinase was detected in plate cultures but not wood cultures, indicating adaption of the fungus to a variety of environmental growth conditions. The high variability of pigmentation in wood cultures suggests that growth of *S. ganodermophthorum* on liquid media and use of extracted pigment is a superior method for obtaining consistent yellow coloration.

## 1. Introduction

*Scytalidium ganodermophthorum* (telomorph: *Xylogone ganodermopthora*) Kang, Sigler, Lee & Yun is a destructive fungal pathogen found on commercial *Ganoderma lucidum* (reishi/lingzhi) mushrooms that causes yellow rot [1]. The eponymous yellow pigment produced by the fungus and similar pigmenting ascomycetes (“spalting” fungi) have been the focus of research in multiple avenues of sustainable natural product development. Wood colorization using *S. ganodermophthorum* has been a particular subject of research, with methods including pressure treatment [2], extracted pigment application [3], oil application [4], and as a component of wood stabilizers [5]. Other investigated pigment uses have included textile dying [6,7,8], use in paints [9], and as a component of organic electronics [10].

The yellow pigment produced by *S. ganodermophthorum* is likely involved with the pathogenicity of the fungus. Filamentous fungi are known to produce pigments involved with pathogenicity [11,12,13,14], and the authors postulated the toxicity of the yellow pigment against host species when the species was described [1]. Work from those authors referenced earlier showed inhibition of *Ganoderma lucidum* growth by the pigment without the presence of the fungus [15], and later demonstrated its anti-fungal activity against a watermelon pathogen [16].

Despite these indications of its biological role, the production of pigment has primarily been accomplished by growing the fungus on wood chip amended media using methods established by Robinson et al. [17] for spalting fungi. This growth method was based on pigment production by related fungi that are capable of soft rot decay of wood: *Scytalidium cuboideum* (Sacc. & Ellis) Sigler & Kang [18] and *Chlorociboria aeruginosa* (Oeder) Seaver ex C.S. Ramamurthi, Korf & L.R. Batra [19]. It is likely that *S. ganodermophthorum* is also capable of soft rot, since in addition to close phylogenic relationships to these species, parasitism has also been seen in the soft rotting fungi *Scytalidium cuboideum* [20] and suggested in *Scytalidium lignicola* [21]. Both species produce type-2 soft rot in wood, characterized by the erosion of the lumen to middle lamella of wood secondary cell walls [18,21,22,23,24]. Adaption to multiple ecological roles may be common among these species.

Changes in metabolic regulation would be expected between fungi engaging in parasitism using a bioactive pigment and those degrading lignocellulosic substrate; thus, pigment production may be impacted by growth on wood. Previous work on soft rot fungi has emphasized the use of additional nutrients to allow for detectible levels of soft rot [25]; however, previous work on spalting fungi has shown that growth on more nutrient-rich media can lead to a loss of pigmentation [26]. Additionally, growth on different wood species produces variable colorations in *S. ganodermophthorum* [27] and shows different decay patterns among other soft rot species [18]. Understanding the balance between enzymatic production for decay and production of potentially bioactive pigments will aid in further work using *S. ganodermophthorum* as a sustainable colorant and shed light on wood-inhabiting pigmenting ascomycete biology.

## 2. Materials and Methods

### 2.1. Block Decay Test

Modified methods following the soft rot decay testing described in Worrall et al. [18] were used to assess mass loss of test blocks, followed by block color analysis. This method was used instead of the standard E-10 American Wood Preservers Association standard [28] for decay testing, which has been shown to be inappropriate for soft-rot decay species [29]. Defect-free, kiln-dried sapwood lumber was used to prepare 14 mm × 14 mm cubes of sugar maple (*Acer saccharum* Marsh), big leaf maple (*Acer macrophyllum* Pursh), Oregon white oak (*Quercus garryana* Douglas ex. Hook), and Douglas fir (*Pseudotsuga menziesii* Mirb). Douglas fir, big leaf maple, and white oak were selected based on their commercial production in Oregon, with white oak additionally included as *S. ganodermophthorum* was initially isolated from *Ganoderma lucidum* cultivation on oak logs [1]. Sugar maple was also tested as *S. ganodermophthorum* has previously shown strong pigment production on this species [27]. 

The prepared cubes were oven-dried for 12 h at 103 °C and the initial dry weight was recorded before half were vacuum infiltrated with either deionized water or nutrient solution consisting of 2 g NH_4_NO_3_, 2.5 g KH_2_PO_4_, 2 g K_2_HPO_4_, 1 g MGSO_4_·7H_2_O, and 2.5 g glucose per liter [25]. In total, 216 blocks per species were prepared to test fungal growth of two strains in high and low nutrient conditions across three time points. The blocks were then buried in 16 oz wide-mouth mason jars containing 15 g vermiculite moistened with either 90 mL of distilled water or nutrient solution, with the top wood face level with the vermiculite surface. Three blocks were placed in each jar. The jars were sterilized with one hour of autoclaving at 121 °C and 21 PSI. 

The test blocks were inoculated with four mm diameter plugs of *S. ganodermophthorum* cultures cut from growth margins with one plug placed on the top surface of each block. Two strains from the University of Alberta Microbial Herbarium were used, UAMH 10320 and UAMH 10321, both originally isolated from oak logs used for mushroom cultivation in Gyeonggi Province, South Korea. Cultures were maintained on 2% malt extract agar (VWR) and grown at room temperature before wood block inoculation. Five jars were inoculated per test condition, with an additional jar as a control. The jars were parafilmed and grown in humidity- and temperature-controlled incubation chambers at 27 ± 2 °C with 80 ± 5% humidity. The jars were collected at 6, 12, and 18 weeks and blocks cleaned of external vermiculite and mycelium using a soft bristled brush. The blocks were oven dried for 12 h at 103 °C and weighed with the loss in dry weight calculated as a percent of the original dry weight.

Pigmentation was assessed following the methods in Vega Gutierrez et al. [30]. The cross-section face of dried, cleaned blocks was color read using a Konica Minolta Chroma Meter CR-5 color reader using Spectramagic NX Color Data software. Values in the CIE L*a*b color space were used to calculate color change along each axis by subtracting average control values from tested condition values. Welch’s ANOVA tests followed by Tukey’s HSD were performed by comparing inoculated to control blocks broken down into individual conditions, as the variation was too great to use standard ANOVA/MANOVA analysis to compare between multiple groups. Analyses were performed using R statical software [31] with the package rstatx [32] and with rgl [33] used for visualization of color data.

### 2.2. Microscopic Examination of Decay

A rapid decay test was performed based on methods described in Leightley [34]. Microtomed sections, roughly 10 mm × 40 mm wide and 12 μm thick, of aspen (*Populus tremuloides* Michx.) were prepared from radial, tangential, and transverse faces of wood and steam sterilized for 20 min at 121 °C. Aspen was selected for this test due to its high decay susceptibility [35]. Growth plates were prepared by pouring roughly 20 mL of 1.5% water-agar in a nine cm Petri plate, then laying sterilized glass rods 1 mm in diameter in the center after cooling. Microtomed sections were laid across the glass supports longitudinally, and then sufficient water-agar was poured on the plate surface to fix the sections in place. Sections were individually inoculated at the point of wood embedment into the agar, and then the plates were parafilmed and allowed to grow for ten weeks. Both *S. ganodermophthorum* strains previously used for block testing were used for inoculation. After ten weeks, the sections were aseptically removed and inspected for evidence of decay with a Nikon Eclipse Ni-U equipped with a Nikon DS-Ri2 camera (Nikon Instruments Inc., Melville, NY, USA).

### 2.3. Enzymatic Analysis

An additional block decay test was prepared as described above, with the modification of using three jars containing one sugar maple block each per species and a growth time of five months. Three plate cultures per species of 1.5% malt–agar media (15 g VWR bacteriological malt and 15 g VWR agar per L of deionized water) were also prepared and allowed to grow over a four-month period. Inoculated blocks, plates, and controls of uninoculated wood blocks and malt–agar plates were used to extract proteins and crude enzyme mixtures. The extraction procedure was adapted from Megazyme endocellulase “CellG5 Method” assay kits (Megazyme, Bray Business Park, Bray Co., Wicklow, Ireland) [36].

Inoculated wood blocks, unexposed control wood blocks, and 5 g of inoculated agar controls were sliced by hand into fine fragments and placed into 35 mL of sterile acetate buffer solution (sodium acetate buffer, 100 mM, pH 4.5 containing 1 mg/mL BSA and 0.02% sodium azide), and stirred at 4 °C.

After 24 h of stirring, the acetate buffer was collected from the samples, which were refilled with fresh acetate buffer and allowed to stir for another 24 h before final collection. The collected acetate buffer was centrifuged at 4 °C for 30 min at 87,000 RCF (28,000 rpm × 10 cm rotor). The supernatant was poured off and stored briefly at 4 °C before being concentrated. Enzyme extracts were concentrated in 15 mL increments at 4 °C at 5000 RCF using Vivaspin-20 centrifugal concentrators (Vivaproducts, Inc., 521 Great Road, Littleton, MA, USA). A volume of approximately 50 to 60 mL was concentrated completely and resuspended in 1.5 mL of citrate buffer (50 mM citric acid adjusted to pH 5.5).

The protein concentrations of the resuspended enzyme extracts were determined by Bradford assay using Coomassie brilliant blue G-250 and standard curves using bovine serum albumin (BSA) [37]. Coomasie dye was read at 595 nm after 10 min at 22 °C, then the extracts were standardized to 20 μg/mL in citrate buffer, and stored at −20 °C. 

All enzyme reactions were carried out in triplicate in 96-well plates, and scanned using a BioTek Epoch2 microplate spectrophotometer (Agilent, 5301 Stevens Creek Blvd., Santa Clara, CA, USA). Blanks for each sample were made using all components of a regular reaction with stop solution added first to pre-stop enzyme activity.

Standardized enzyme extracts each containing an equivalent protein concentration of 20 μg/mL were assayed for cellulase activity using Megazyme CellG5 kits with procedures modified to use 96-well plates.

In short, 195 μL reaction volumes contained 0.2 μg of crude enzyme protein, 10 μL of prepared para-nitrophenol bound cellulose substrate, 4,6-O-(3-Ketobutylidene)-4-nitrophenyl-β-d-cellopentaoside (BPNPG5) in 10% DMSO/H2O, and 50 mM citrate buffer (pH 5.5) solution. The enzyme reactions were incubated for periods of 10 min, 20 min, and 60 min at 40 °C, then stopped by the addition of 155 μL of 1.0 mM NaOH (buffered to pH 13). The molar concentration of released nitrophenol was read at 405 nm and compared to a standard curve of nitrophenol in 1 mM NaOH stop solution. Higher volumes of extract were also used, but suffered increasing interference from yellow pigment in some samples. The substrate for endo-1,4-β-d-glucanase was validated by testing with Trichoderma purified cellulase supplied with the Megazyme kit. 

Chitinase activity was assessed similarly by 195 μL reactions containing 0.2 μg of enzyme extract, 512.82 μM 4-Nitrophenyl N-acetyl-β-d-glucosaminide, (Sigma-Aldrich, St. Louis, MO, USA), and 50 mM citrate buffer (pH 5.5). The reactions were stopped after 10, 20, and 60 min of incubation at 37 °C by the addition of 155 μL of 1.0 mM NaOH (buffered to pH 13) for a resulting final concentration of 0.44 mM. The final concentration of the substrate was 285.7 μM.

Enzyme activity for β-N-acetylglucosaminidase (chitinase) increased with time, and the rate of substrate conversion was calculated at 60 min. Enzyme activity for endo-1,4-β-d-glucanase was low to absent at all time points but was calculated from the change in absorbance at 60 min. Enzyme relative activity is reported as μM 4-nitrophenol evolved per minute per ug enzyme extract.

## 3. Results

### 3.1. Block Color Change Analysis

**Figure 1:**
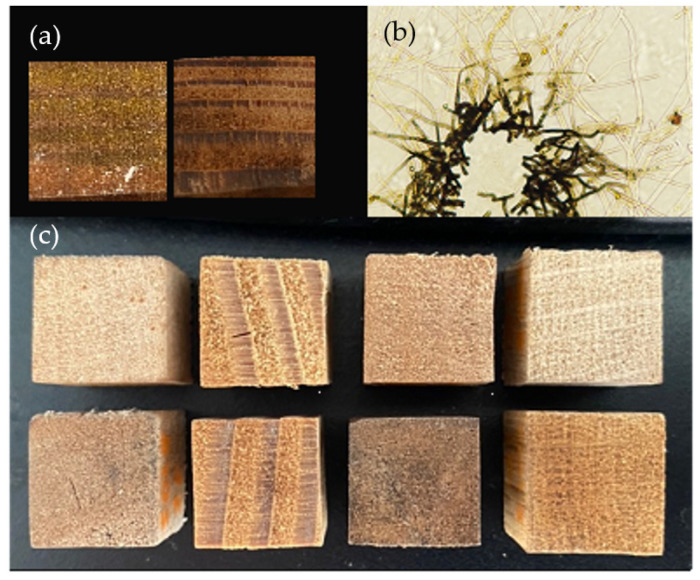
Pigmentation produced by *Scytalidium ganodermophthorum*. (**a**) Two nutrient-supplemented Douglas fir blocks colonized by *S. ganodermophthorum* strain UAMH 10321 and incubated for 18 weeks. Left block shows deposition of a yellow pigment, while the right shows dark pigmentation. (**b**) Light microscopy of *S. ganodermophthorum* hyphae showing both yellow and dark pigments. (**c**) Comparison between control blocks (top row) and inoculated blocks showing the strongest yellow coloration as measured by b* color value (bottom row) for each wood species.

The inoculated wood blocks showed variable coloration between and within tested conditions. Both the expected yellow coloration and darker colors were produced in the blocks without consistency between wood species, strain, or nutrient supplementation groups (Figure 1a). These pigments could also be seen in plate culture, with the dark pigment proximal to plate inoculation points and yellow pigmented vesicles present in hyphae radiating out (Figure 1b). Visual examination showed clear differences between the inoculated blocks showing the strongest yellow pigmentation (highest b* value in L*a*b* color space) and control blocks for each wood species (Figure 1c). 

**Figure 2:**
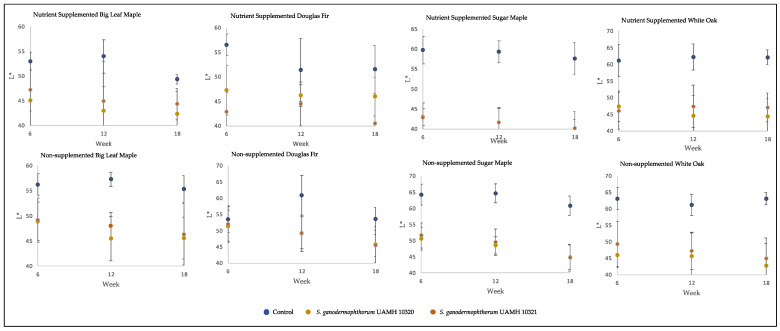
Block L* values across tested conditions. Low L* values represent darker colors, with high L* values representing brighter colors. Significant differences were seen between control and both *S. ganodermophthorum* strain values in all conditions except week six nutrient-supplemented Douglas fir and, with week six nutrient-supplemented sugar maple showing additional significant differences between strains (Tables S2 and S3).

The inoculated blocks showed darker coloration than control, with L* values significantly lower than those of control (*p* < 0.5) in all but two conditions (Figure 2). Values along the a* and b* axes, representing (+) red to (−) green and (+) yellow to blue (−) colors, respectively, were not so consistent. At the longest incubation time point, the only significant difference from control for both strains along the a* axis was seen in nutrient-supplemented white oak, representing a redder coloration (F = 48.73, *p* = 8.9 × 10^−9^). The b* axis showed significantly lower values than control (*p* < 0.5, Table S1) in nutrient-treated sugar maple and untreated maple species and white oak, representing shifts towards blue.

**Figure 3:**
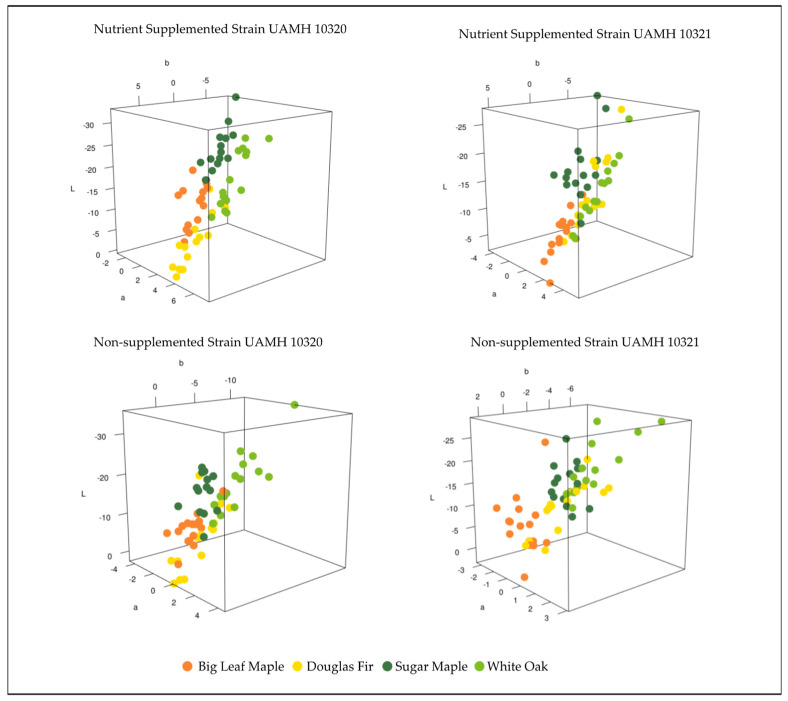
Color difference in L*a*b* color space for inoculated wood species vs. control. Wood species demonstrated varying levels of clustering between fungal strains and nutrient conditions. Nutrient conditions exhibited more structured color changes, appearing to follow a pattern of change towards dark green/blue (low/negative L*, a*, and b* values) or lighter red/yellow (higher L*, a*, and b* values). In general, Douglas fir and big leaf maple blocks appeared more yellow than controls compared to sugar maple and white oak.

**Figure 4:**
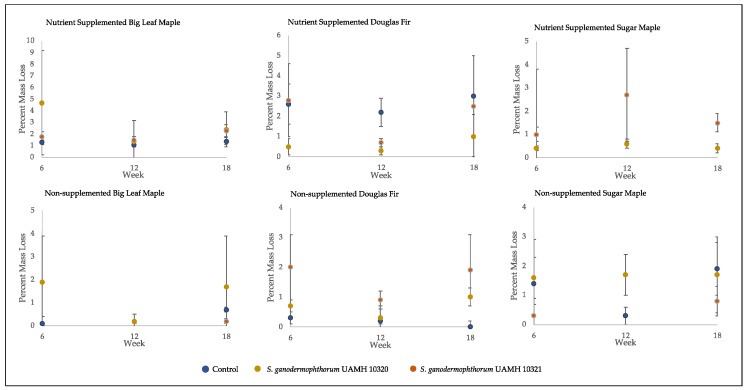
Mass loss of decay test blocks. Nutrient-supplemented Doug fir blocks were significantly different than control for both strains across all weeks (*p* > 0.5, Tables S4 and S5). A significant difference was also seen between inoculated blocks and control in non-supplemented sugar maple for week 12 blocks; however, this difference was not seen at week 18. Overall mean values were around 2% mass loss, with high variability seen across conditions.

Comparing the color differences in L*a*b* three-dimensional color space between control and inoculated blocks at week 18 for each wood species showed patterns between strain and nutrient supplementation (Figure 3). Strain UAMH 10320 in nutrient-supplemented conditions showed that colors clustered by species along an apparent trend of shifts towards dark blue/green (low/negative L*, a*, and b* values) or lighter yellow/red (higher L*, a*, and b* values) (Figure 4). This is likely associated with the production of more yellow pigment or dark pigment depending on the wood species. Alignment along this trend could also be seen in nutrient-supplemented strain UAMH 10321 and non-supplemented strain UAMH 10320, although these showed more variability resulting in looser species clusters. 

### 3.2. Assessment of Wood Decay Capabilities

The average percent mass loss of *Scytalidium ganodermophthorum*-treated wood blocks was extremely variable (Figure 4). The blocks incubated for 18 weeks showed no statistically significant differences between any tested conditions and control, with average mass loss values of around two percent, which is considered a threshold for decay [18].

**Figure 5:**
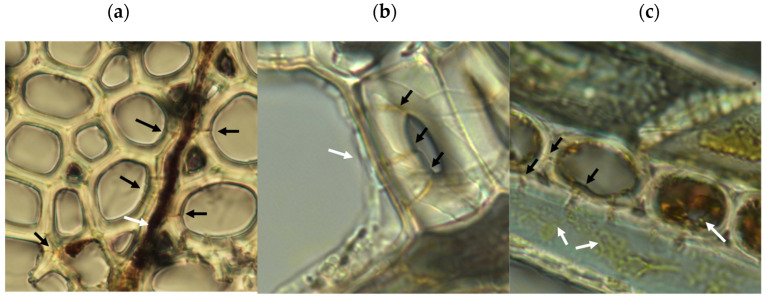
Microscopic evidence of soft rot decay in aspen from rapid decay testing. Black arrows show boreholes, white arrows show fungal hyphae. (**a**) Transverse wood section showing boreholes through wood fiber cell walls adjacent to a colonized wood ray, with angular edges seen in cell lumens. (**b**) Hyphal growth in cell lumen showing evidence of cell penetration and boreholes. (**c**) Tangential wood section showing yellow pigmented hyphae in wood fiber, with angular notches in fiber walls characteristic of type-2 soft rot. Internal erosion of ray cell lumen and lack of cell contents indicate cellular digestion. Ray cell adjacent to the emptied cell shows internal hyphae proximal to reduced wood ray cell contents. For scale, wood cells in the sample averaged approximately 20 μm in diameter.

Microscopic analysis of samples from the rapid decay test showed evidence of type-2 soft rot (Figure 5). Transverse boreholes were present with angular V-shaped notches in lumen walls indicating structural erosion, features characteristic of type-2 soft rot [18,38]. The type 1 soft rot characteristics of diamond-shaped holes and cavitation of the S2 layer of wood cell walls were not seen. This production of type-2 but not type-1 soft rot is consistent with the characteristics of the closely related species *Scytalidium cuboideum* and *Scytalidium lignicola* [18].

**Figure 6:**
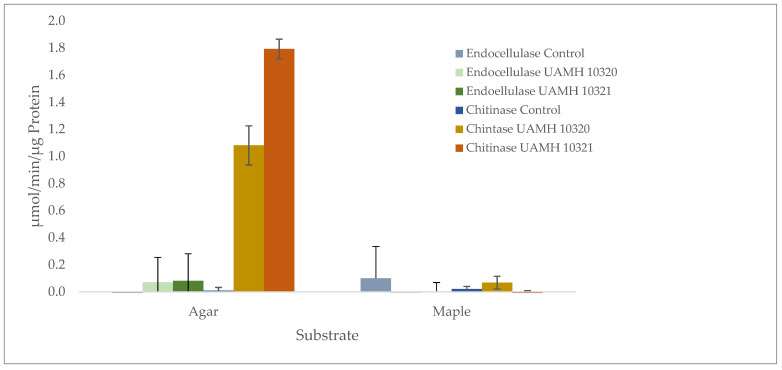
Presence of endoglucanase and chitinase activity in agar and maple substrates inoculated with *S. ganodermopthorum*. Low levels of endocellulase activity were detected across conditions, with no significant difference from uninoculated control blocks or agar (F = 1.133, *p* = 0.354). Chitinase activity was significantly higher (F = 251.71, *p* = 1.59 × 10^−10^) in agar cultures, with Tukey’s comparisons showing differences in chitinase activity between both strains and other tested conditions (Tables S6 and S7).

Enzymatic analysis showed no detectible endocellulase activity that was significantly different from uninoculated wood or agar controls. However, chitinase activity was detected in agar plates of both strains well above controls (Figure 6). Enzyme-specific activity varied between strains, indicating high biological variability between cultures. 

## 4. Discussion

The pigmentation and decay by *Scytalidium ganodermophthorum* were highly variable. Many blocks showed the expected yellow coloration; however, others exhibited a dark pigment. Previous work has shown that yellow coloration is produced by these fungal strains grown in highly similar conditions on sugar maple, but did not report the presence of the darker pigment in wood [27]. This may indicate changes in gene expression due to culture age or other environmental conditions. The dark coloration seen in the wood is likely from a colored compound or secondary pigment that appears green or purple depending on pH [39]. Extraction of colonized wood chip plates over multiple time points have shown a color shift of pigmented extracts changing from yellow to green to purple over time [27]. This is consistent with culture examinations where older growth showed darker pigmentation. 

Microscopic assessment of decay confirmed that *S. ganodermophthorum* can produce type-2 soft rot in wood; however, block decay tests showed weak degradation and high variability. Related pigmenting ascomycete soft rot species have shown mass loss values substantially higher than the 2% range seen here, with *S. cuboideum* showing a 9.6% mean mass loss and *S. lignicola* showing a 6.5% mean mass loss over a 12-week period in birch [18]. While differences in decay capabilities of hard and softwoods are expected from fungi, and soft wood decay has been shown to be lower in *S. cuboideum* and *S. lignicola* grown on pine (2.4%), the range of values seen between Douglas fir, big leaf maple, and sugar maple were similar. While supplementation of nutrients was previously described as critical for detection of soft rot in block decay tests [25], no differences could be perceived in these results.

Chitinase production is consistent with a mycoparasitic lifestyle, and previous descriptions of cultures include the capacity to lyse cell walls [1]. The lack of detectible chitinase activity higher than control on a woody substrate indicates the shifting of fungal metabolism in response to environmental conditions. Endocellulase production would have been expected in conjunction with evidence of soft rot decay but was not present at detectible levels. Cellulose- and hemicellulose-degrading enzymes are widely active in soft rot fungi [23,40], and can be either secreted or within cells [41,42], and have been speculated to be important for cell cavity widening [43]. Lack of cellulase activity despite the observation of cell wall modifications could be the result of several factors. Highly decay-susceptible aspen was used for microscopic decay examination, while enzyme analysis was performed on sugar maple blocks that could have had reduced decay activity. Other carbohydrate-degrading enzymes known to be produced by soft rot could also be taking on a larger role in cell wall degradation by *S. ganodermophthorum* [44]. These could be mannases and xylanases [40], or potentially exocellulases which would not have been detected with our methods. 

Additionally, it is possible that endoglucanase activity had occurred during earlier stages of wood colonization not tested here. Boreholes were observed in wood colonized by *S. ganodermophthorum* after ten weeks and enzymes were extracted from wood that had been degraded for five months. It is possible that limited cellulolysis occurred in the early stages of decay during substrate colonization by *S. ganodermophthorum*, which had subsided by the time enzyme extracts were made for this study. In both white and brown rot decay fungi, gene expression and protein secretion profiles can change dramatically even over the course of a few days or weeks during the decay process [45]. A similar temporal shift in protein secretion could also be at play here, but without further study this is only speculative.

The decay capabilities demonstrated here may have implications for pathogen management, as growth in wood may serve as a reservoir for disease. Its mycoparasitic capabilities may also indicate its use as a biocontrol agent against plant pathogenic fungi, as has been shown in the closely related *Scytalidium parasiticum* (Yit K. Goh, Goh, Y.K. Goh & K.J. Goh) [46,47] and *Trichoderma* species [48]. *Scytalidium ganodermophthorum* was also recently described as an endophyte in plant roots, indicating its adaption to a range of growth conditions outside of mycoparasitism [49]. Other *Scytalidium* species have also been reported to infect skin and nails [50,51] and as plant pathogens [52,53]. The range of lifestyles seen within the genus and versatility seen in *S. ganodermophthorum* indicate that they have strategies for adaptive flexibility vs. targeted adaption to a specific lifestyle. 

Due to high levels of within-group pigmentation and decay results, no relationship could be drawn between pigment production and degree of wood decay. However, lack of structural decay and the previously demonstrated antifungal properties show potential for the use of *S. ganodermophthorum* as a pretreatment or the extracted pigment as an antifungal agent for wood treatment. However, there are indications of potential environmental toxicity [54], so care must be taken in product development. The high variability of pigment production indicate that liquid cultures may be a superior method of pigment production for wood coloration or other applications. 

## Data Availability

The data presented in this study are available on request from the corresponding author.

## Supplementary Materials

The following supporting information can be downloaded at: https://www.mdpi.com/article/10.3390/jof9070738/s1, Table S1. Welch’s ANOVA test for week 18 color difference values. Table S2. Welch’s ANOVA test for ΔL color differences across weeks. Table S3. Tukey’s HSD comparisons for ΔL* values across weeks. Table S4. Welch’s ANOVA test results for percent mass loss by week. Table S5. Tukey’s HSD comparisons of block conditions and percent mass loss across weeks. Table S6. ANOVA test results for enzyme activity. Table S7. Tukey’s HSD test for ANOVA results for enzyme activity.
