# Supplementary material for: Wood Coloration and Decay Capabilities of Mycoparasite Scytalidium ganodermophthorum
Source: J Fungi (Basel). 2023 Jul 11;9(7):738. doi: 10.3390/jof9070738 (PMC10381127; doi:10.3390/jof9070738)
Supplement: Supplementary file 1 [file jof-09-00738-s001.zip › jof-2428230-supplementary.pdf]

Journal of *Fungi*

MDPI

Supplemental Materials.

Table S1. Welch's ANOVA test for week 18 color difference values.

Week	Nutrients	Wood	n	Statistic	Dfn	Dfd	p- value
ΔL	Nutrient supplemented	Douglas fir	36	21.35	2	15.14471	3.92E-05
ΔL	Nutrient supplemented	Big leaf maple	36	27.18	2	21.85131	1.18E-06
ΔL	Nutrient supplemented	Sugar maple	36	48.69	2	14.39766	3.91E-07
ΔL	Nutrient supplemented	White oak	36	79.91	2	19.92749	3.04E-10
ΔL	Non-supplemented	Douglas fir	36	15.13	2	14.22267	0.000301
ΔL	Non-supplemented	Big leaf maple	36	22.3	2	18.61597	1.14E-05
ΔL	Non-supplemented	Sugar maple	36	92.27	2	18.30766	2.74E-10
ΔL	Non-supplemented	White oak	36	92.11	2	21.96461	2.09E-11
ΔA	Nutrient supplemented	Douglas fir	36	11.63	2	21.52697	0.000377
ΔA	Nutrient supplemented	Big leaf maple	36	1.54	2	13.61946	0.249
ΔA	Nutrient supplemented	Sugar maple	36	0.2	2	20.04202	0.824
ΔA	Nutrient supplemented	White oak	36	48.73	2	21.83484	8.88E-09
ΔA	Non-supplemented	Douglas fir	36	1.69	2	17.09425	0.213
ΔA	Non-supplemented	Big leaf maple	36	0.67	2	14.36269	0.528
ΔA	Non-supplemented	Sugar maple	36	6.31	2	16.08743	0.009
ΔA	Non-supplemented	White oak	36	2.15	2	21.76089	0.141
ΔB	Nutrient supplemented	Douglas fir	36	9.22	2	17.73677	0.002
ΔB	Nutrient supplemented	Big leaf maple	36	1.18	2	15.60035	0.334
ΔB	Nutrient supplemented	Sugar maple	36	4.55	2	18.3074	0.025
ΔB	Nutrient supplemented	White oak	36	1.67	2	21.92943	0.212
ΔB	Non-supplemented	Douglas fir	36	1.61	2	18.27395	0.227
ΔB	Non-supplemented	Big leaf maple	36	3.93	2	16.84513	0.04
ΔB	Non-supplemented	Sugar maple	36	6.03	2	13.3151	0.014
ΔB	Non-supplemented	White oak	36	18.73	2	14.6492	9.20E-05

Table S2. Welch's ANOVA test for ΔL color differences across weeks

Week	Nutrients	Wood	n	Statistic	Dfn	Dfd	p-value
**18**	Nutrient supplemented	Douglas fir	36	10.85	2	13.6463	0.002
**18**	Nutrient supplemented	Big leaf maple	36	27.3	2	21.84226	1.15E-06
**18**	Nutrient supplemented	Sugar maple	36	49.4	2	14.61064	3.15E-07
**18**	Nutrient supplemented	White oak	36	81.55	2	20.08609	2.28E-10
**18**	Non-supplemented	Douglas fir	36	12.23	2	14.81172	0.000732
**18**	Non-supplemented	Big leaf maple	36	22.22	2	18.6073	1.17E-05
**18**	Non-supplemented	Sugar maple	36	61.18	2	15.95086	3.30E-08
**18**	Non-supplemented	White oak	36	92.11	2	21.96461	2.09E-11
**12**	Nutrient supplemented	Douglas fir	36	2.84	2	11.29606	0.1
**12**	Nutrient supplemented	Big leaf maple	36	17.85	2	18.0774	5.25E-05
**12**	Nutrient supplemented	Sugar maple	36	87.4	2	17.15049	1.01E-09
**12**	Nutrient supplemented	White oak	36	34.44	2	17.43424	8.80E-07
**12**	Non-supplemented	Douglas fir	36	8.95	2	13.45056	0.003
**12**	Non-supplemented	Big leaf maple	36	71.99	2	20.35771	5.86E-10
**12**	Non-supplemented	Sugar maple	36	68.23	2	14.32112	4.78E-08
**12**	Non-supplemented	White oak	36	35.73	2	19.52813	2.98E-07
**6**	Nutrient supplemented	Douglas fir	36	51.03	2	19.49164	1.79E-08
**6**	Nutrient supplemented	Big leaf maple	36	15.5	2	21.34862	6.96E-05
**6**	Nutrient supplemented	Sugar maple	36	61.66	2	12.49335	3.36E-07
**6**	Nutrient supplemented	White oak	36	21.1	2	14.25719	5.49E-05
**6**	Non-supplemented	Douglas fir	36	0.51	2	15.68529	0.611
**6**	Non-supplemented	Big leaf maple	36	18.76	2	19.00405	3.17E-05

**6**	Non-supplemented	Sugar maple	36	37.34	2	14.96449	1.52E-06
**6**	Non-supplemented	White oak	36	50.05	2	15.87426	1.40E-07

Table S3. Tukey's HSD comparisons for ΔL* values across weeks

Week	Nutrients	Wood Species	Condition 1	Condition 2	Low Confidence	High Confidence	Adjusted p-value	Significance
**18**	Nutrient supplemented	Doug fir	Control	Sg_0	-3.77533	-9.15185	1.601185	2.12E-01
**18**	Nutrient supplemented	Doug fir	Control	Sg_1	-12.814	-18.1905	-7.43748	4.44E-06
**18**	Nutrient supplemented	Doug fir	Sg_0	Sg_1	-9.03867	-13.1029	-4.9744	1.40E-05
**18**	Nutrient supplemented	Big leaf maple	Control	Sg_0	-7.06533	-11.3367	-2.79395	0.000812
**18**	Nutrient supplemented	Big leaf maple	Control	Sg_1	-4.994	-9.26538	-0.72262	0.019
**18**	Nutrient supplemented	Big leaf maple	Sg_0	Sg_1	2.071333	-1.15753	5.300192	0.271
**18**	Nutrient supplemented	Sugar maple	Control	Sg_0	-20.7618	-25.9189	-15.6048	6.56E-11
**18**	Nutrient supplemented	Sugar maple	Control	Sg_1	-16.4348	-21.5919	-11.2778	1.54E-08
**18**	Nutrient supplemented	Sugar maple	Sg_0	Sg_1	4.327	0.428617	8.225383	2.70E-02
**18**	Nutrient supplemented	White oak	Control	Sg_0	-17.6252	-23.0146	-12.2357	8.73E-09
**18**	Nutrient supplemented	White oak	Control	Sg_1	-15.2068	-20.5963	-9.8174	1.94E-07
**18**	Nutrient supplemented	White oak	Sg_0	Sg_1	2.418333	-1.6557	6.492363	3.25E-01
**18**	Non-supplemented	Doug fir	Control	Sg_0	-6.1	-11.4296	-0.77044	0.022

**18**	Non-supplemented	Doug fir	Control	Sg_1	-9.882	-15.2116	-4.55244	0.0002
**18**	Non-supplemented	Doug fir	Sg_0	Sg_1	-3.782	-7.81077	0.24677	0.0693
**18**	Non-supplemented	Big leaf maple	Control	Sg_0	-9.77533	-15.6325	-3.9182	0.000732
**18**	Non-supplemented	Big leaf maple	Control	Sg_1	-8.962	-14.8191	-3.10487	0.00189
**18**	Non-supplemented	Big leaf maple	Sg_0	Sg_1	0.813333	-3.61424	5.240909	0.894
**18**	Non-supplemented	Sugar maple	Control	Sg_0	-16.9548	-21.3985	-12.5112	2.45E-10
**18**	Non-supplemented	Sugar maple	Control	Sg_1	-15.0625	-19.5061	-10.6189	3.91E-09
**18**	Non-supplemented	Sugar maple	Sg_0	Sg_1	1.892333	-1.46673	5.251392	3.62E-01
**18**	Non-supplemented	White oak	Control	Sg_0	-20.1882	-27.3501	-13.0263	1.98E-07
**18**	Non-supplemented	White oak	Control	Sg_1	-18.2805	-25.4424	-11.1186	1.32E-06
**18**	Non-supplemented	White oak	Sg_0	Sg_1	1.907667	-3.50623	7.32156	6.66E-01
**12**	Nutrient supplemented	Doug fir	Control	Sg_0	-5.181	-10.0612	-0.30082	0.0355
**12**	Nutrient supplemented	Doug fir	Control	Sg_1	-6.903	-11.7832	-2.02282	0.00408
**12**	Nutrient supplemented	Doug fir	Sg_0	Sg_1	-1.722	-5.41107	1.967068	0.494
**12**	Nutrient supplemented	Big leaf maple	Control	Sg_0	-10.989	-18.3804	-3.59756	0.00253
**12**	Nutrient supplemented	Big leaf maple	Control	Sg_1	-9.08433	-16.4758	-1.69289	0.0132
**12**	Nutrient supplemented	Big leaf maple	Sg_0	Sg_1	1.904667	-3.68274	7.49207	0.683

**12**	Nutrient supple-mented	Sugar maple	Control	Sg_0	-20.2303	-25.9698	-14.4908	1.60E-09
**12**	Nutrient supple-mented	Sugar maple	Control	Sg_1	-17.6543	-23.3938	-11.9148	3.29E-08
**12**	Nutrient supple-mented	Sugar maple	Sg_0	Sg_1	2.576	-1.76265	6.91465	3.24E-01
**12**	Nutrient supple-mented	White oak	Control	Sg_0	-10.989	-18.3804	-3.59756	0.00253
**12**	Nutrient supple-mented	White oak	Control	Sg_1	-9.08433	-16.4758	-1.69289	0.0132
**12**	Nutrient supple-mented	White oak	Sg_0	Sg_1	1.904667	-3.68274	7.49207	0.683
**12**	Non-sup-ple-mented	Doug fir	Control	Sg_0	-11.6437	-18.0521	-5.23527	0.00026
**12**	Non-sup-ple-mented	Doug fir	Control	Sg_1	-11.5523	-17.9607	-5.14394	0.000287
**12**	Non-sup-ple-mented	Doug fir	Sg_0	Sg_1	0.091333	-4.75296	4.935624	0.999
**12**	Non-sup-ple-mented	Big leaf maple	Control	Sg_0	-11.8233	-15.8536	-7.79306	8.84E-08
**12**	Non-sup-ple-mented	Big leaf maple	Control	Sg_1	-9.314	-13.3443	-5.28373	7.48E-06
**12**	Non-sup-ple-mented	Big leaf maple	Sg_0	Sg_1	2.509333	-0.53727	5.555935	1.23E-01
**12**	Non-sup-ple-mented	Sugar maple	Control	Sg_0	-16.0977	-20.1027	-12.0926	6.83E-11
**12**	Non-sup-ple-mented	Sugar maple	Control	Sg_1	-15.103	-19.1081	-11.0979	3.25E-10
**12**	Non-sup-ple-mented	Sugar maple	Sg_0	Sg_1	0.994667	-2.03288	4.022216	7.02E-01
**12**	Non-sup-ple-mented	White oak	Control	Sg_0	-15.5493	-22.6745	-8.42412	1.90E-05
**12**	Non-sup-ple-mented	White oak	Control	Sg_1	-14.012	-21.1372	-6.88679	8.95E-05

**12**	Non-supplemented	White oak	Sg_0	Sg_1	1.537333	-3.84882	6.923489	7.65E-01
**6**	Nutrient supplemented	Doug fir	Control	Sg_0	-9.211	-14.2626	-4.15937	2.48E-04
**6**	Nutrient supplemented	Doug fir	Control	Sg_1	-13.6263	-18.678	-8.57471	4.67E-07
**6**	Nutrient supplemented	Doug fir	Sg_0	Sg_1	-4.41533	-8.234	-0.59666	2.05E-02
**6**	Nutrient supplemented	Big leaf maple	Control	Sg_0	-7.925	-13.7059	-2.14412	0.00542
**6**	Nutrient supplemented	Big leaf maple	Control	Sg_1	-5.79233	-11.5732	-0.01145	0.0495
**6**	Nutrient supplemented	Big leaf maple	Sg_0	Sg_1	2.132667	-2.23727	6.502601	0.463
**6**	Nutrient supplemented	Sugar maple	Control	Sg_0	-16.7243	-20.3276	-13.1211	1.76E-12
**6**	Nutrient supplemented	Sugar maple	Control	Sg_1	-16.8983	-20.5016	-13.2951	1.35E-12
**6**	Nutrient supplemented	Sugar maple	Sg_0	Sg_1	-0.174	-2.89781	2.549808	9.87E-01
**6**	Nutrient supplemented	White oak	Control	Sg_0	-13.7903	-19.7175	-7.86314	6.68E-06
**6**	Nutrient supplemented	White oak	Control	Sg_1	-15.1937	-21.1209	-9.26647	1.22E-06
**6**	Nutrient supplemented	White oak	Sg_0	Sg_1	-1.40333	-5.88387	3.077204	7.25E-01
**6**	Non-supplemented	Doug fir	Control	Sg_0	-2.13933	-8.02868	3.750015	0.649
**6**	Non-supplemented	Doug fir	Control	Sg_1	-1.476	-7.36535	4.413348	0.813
**6**	Non-supplemented	Doug fir	Sg_0	Sg_1	0.663333	-3.7886	5.115262	0.929
**6**	Non-supplemented	Big leaf maple	Control	Sg_0	-7.312	-11.9259	-2.69811	0.00131

**6**	Non-supplemented	Big leaf maple	Control	Sg_1	-7.078	-11.6919	-2.46411	0.00184
**6**	Non-supplemented	Big leaf maple	Sg_0	Sg_1	0.234	-3.25377	3.721774	0.985
**6**	Non-supplemented	Sugar maple	Control	Sg_0	-13.5863	-17.9234	-9.24927	2.23E-08
**6**	Non-supplemented	Sugar maple	Control	Sg_1	-12.6017	-16.9387	-8.26461	1.08E-07
**6**	Non-supplemented	Sugar maple	Sg_0	Sg_1	0.984667	-2.29384	4.263175	7.43E-01
**6**	Non-supplemented	White oak	Control	Sg_0	-17.089	-23.3328	-10.8452	3.53E-07
**6**	Non-supplemented	White oak	Control	Sg_1	-13.7917	-20.0355	-7.54785	1.56E-05
**6**	Non-supplemented	White oak	Sg_0	Sg_1	3.297333	-1.42255	8.017214	2.15E-01

Table S4. Welch's ANOVA test results for percent mass loss by week.

Week	Nutrients	Wood	n	Statistic	Dfn	Dfd	p-value
**18**	Nutrient supplemented	Doug fir	36	48.92	2	13.06143	8.58E-07
**18**	Nutrient supplemented	Big leaf maple	36	18.93	2	19.22165	2.86E-05
**18**	Nutrient supplemented	sugar maple	36	0.05	2	13.3992	0.951
**18**	Non-supplemented	Doug fir	36	3.14	2	11.24719	0.083
**18**	Non-supplemented	Big leaf maple	36	1.11	2	10.7046	0.364
**18**	Non-supplemented	Sugar maple	36	8.7	2	17.45784	0.002
**12**	Nutrient supplemented	Doug fir	36	102.24	2	21.23627	1.26E-11
**12**	Nutrient supplemented	Big leaf maple	36	17.09	2	19.25326	5.40E-05

**12**	Nutrient supple-mented	Sugar maple	36	10.18	2	19.53943	0.000936
**12**	Non-sup-plemented	Doug fir	36	6.14	2	10.8052	0.017
**12**	Non-sup-plemented	Big leaf maple	36	3.56	2	11.17457	0.064
**12**	Non-sup-plemented	Sugar maple	36	0.49	2	17.12183	0.624
**6**	Nutrient supple-mented	Doug fir	36	21.66	2	12.84932	7.66E-05
**6**	Nutrient supple-mented	Big leaf maple	36	11.99	2	19.92528	0.000381
**6**	Nutrient supple-mented	Sugar maple	36	19.03	2	21.94296	1.61E-05
**6**	Non-sup-plemented	Doug fir	36	30.07	2	20.14843	8.95E-07
**6**	Non-sup-plemented	Big leaf maple	36	25.43	2	11.52978	5.92E-05
**6**	Non-sup-plemented	Sugar maple	36	5.45	2	19.22584	0.013

Table S5. Tukey's HSD comparisons of block conditions and percent mass loss across weeks.

Week	Nutrients	Wood Spe-cies	Condi-tion 1	Condi-tion 2	Low Con-fidence	High Confi-dence	Ad-justed p-value	Signifi-cance
**18**	Nutrient supple-mented	Doug fir	Control	Sg_0	0.0069	0.0050	0.0087	4.75E-10
**18**	Nutrient supple-mented	Doug fir	Control	Sg_1	0.0067	0.0048	0.0085	1.01E-09
**18**	Nutrient supple-mented	Doug fir	Sg_0	Sg_1	-0.0002	-0.0016	0.0012	9.24E-01
**18**	Nutrient supple-mented	Big leaf ma-ple	Control	Sg_0	0.0248	0.0009	0.0486	0.0404
**18**	Nutrient supple-mented	Big leaf ma-ple	Control	Sg_1	0.0217	-0.0022	0.0456	0.0804
**18**	Nutrient supple-mented	Big leaf ma-ple	Sg_0	Sg_1	-0.0031	-0.0211	0.0150	0.908

**18**	Nutrient supple-mented	Sugar ma-ple	Control	Sg_0	-0.0003	-0.0214	0.0209	0.999
**18**	Nutrient supple-mented	Sugar ma-ple	Control	Sg_1	0.0018	-0.0194	0.0229	0.977
**18**	Nutrient supple-mented	Sugar ma-ple	Sg_0	Sg_1	0.0020	-0.0140	0.0180	0.948
**18**	Non-sup-ple-mented	Doug fir	Control	Sg_0	0.0047	-0.0134	0.0227	0.804
**18**	Non-sup-ple-mented	Doug fir	Control	Sg_1	0.0066	-0.0115	0.0247	0.648
**18**	Non-sup-ple-mented	Doug fir	Sg_0	Sg_1	0.0019	-0.0117	0.0156	0.936
**18**	Non-sup-ple-mented	Big leaf ma-ple	Control	Sg_0	0.0049	-0.0037	0.0135	0.354
**18**	Non-sup-ple-mented	Big leaf ma-ple	Control	Sg_1	0.0019	-0.0067	0.0105	0.852
**18**	Non-sup-ple-mented	Big leaf ma-ple	Sg_0	Sg_1	-0.0030	-0.0095	0.0035	0.501
**18**	Non-sup-ple-mented	Sugar ma-ple	Control	Sg_0	0.0051	0.0005	0.0097	0.026
**18**	Non-sup-ple-mented	Sugar ma-ple	Control	Sg_1	0.0057	0.0011	0.0103	0.0131
**18**	Non-sup-ple-mented	Sugar ma-ple	Sg_0	Sg_1	0.0005	-0.0030	0.0040	0.927
**12**	Nutrient supple-mented	Doug fir	Control	Sg_0	0.0089	0.0062	0.0115	5.84E-09
**12**	Nutrient supple-mented	Doug fir	Control	Sg_1	0.0077	0.0050	0.0103	1.33E-07
**12**	Nutrient supple-mented	Doug fir	Sg_0	Sg_1	-0.0012	-0.0032	0.0008	3.15E-01
**12**	Nutrient supple-mented	Big leaf ma-ple	Control	Sg_0	0.0219	-0.0105	0.0542	0.236
**12**	Nutrient supple-mented	Big leaf ma-ple	Control	Sg_1	0.0392	0.0069	0.0716	0.0146

**12**	Nutrient supplemented	Big leaf maple	Sg_0	Sg_1	0.0174	-0.0071	0.0418	0.205
**12**	Nutrient supplemented	Sugar maple	Control	Sg_0	0.0368	0.0005	0.0731	0.0464
**12**	Nutrient supplemented	Sugar maple	Control	Sg_1	0.0134	-0.0229	0.0497	0.64
**12**	Nutrient supplemented	Sugar maple	Sg_0	Sg_1	-0.0234	-0.0509	0.0041	0.107
**12**	Non-supplemented	Doug fir	Control	Sg_0	0.0045	-0.0134	0.0224	0.814
**12**	Non-supplemented	Doug fir	Control	Sg_1	0.0023	-0.0157	0.0202	0.949
**12**	Non-supplemented	Doug fir	Sg_0	Sg_1	-0.0022	-0.0158	0.0113	0.915
**12**	Non-supplemented	Big leaf maple	Control	Sg_0	0.0056	0.0009	0.0102	0.0168
**12**	Non-supplemented	Big leaf maple	Control	Sg_1	0.0023	-0.0024	0.0069	0.468
**12**	Non-supplemented	Big leaf maple	Sg_0	Sg_1	-0.0033	-0.0068	0.0002	0.0713
**12**	Non-supplemented	Sugar maple	Control	Sg_0	0.0011	-0.0031	0.0053	0.795
**12**	Non-supplemented	Sugar maple	Control	Sg_1	0.0013	-0.0030	0.0055	0.742
**12**	Non-supplemented	Sugar maple	Sg_0	Sg_1	0.0002	-0.0030	0.0034	0.992
**6**	Nutrient supplemented	Doug fir	Control	Sg_0	0.0071	0.0047	0.0095	6.61E-08
**6**	Nutrient supplemented	Doug fir	Control	Sg_1	0.0050	0.0026	0.0074	4.09E-05
**6**	Nutrient supplemented	Doug fir	Sg_0	Sg_1	-0.0022	-0.0040	-0.0003	1.68E-02
**6**	Nutrient supplemented	Big leaf maple	Control	Sg_0	0.0209	-0.0025	0.0442	0.0873

**6**	Nutrient supplemented	Big leaf maple	Control	Sg_1	0.0191	-0.0043	0.0424	0.127
**6**	Nutrient supplemented	Big leaf maple	Sg_0	Sg_1	-0.0018	-0.0194	0.0159	0.966
**6**	Nutrient supplemented	Sugar maple	Control	Sg_0	0.0166	0.0038	0.0295	0.00884
**6**	Nutrient supplemented	Sugar maple	Control	Sg_1	0.0161	0.0033	0.0290	0.0113
**6**	Nutrient supplemented	Sugar maple	Sg_0	Sg_1	-0.0005	-0.0102	0.0092	0.991
**6**	Non-supplemented	Doug fir	Control	Sg_0	0.0031	0.0016	0.0047	7.32E-05
**6**	Non-supplemented	Doug fir	Control	Sg_1	0.0027	0.0011	0.0042	6.12E-04
**6**	Non-supplemented	Doug fir	Sg_0	Sg_1	-0.0005	-0.0017	0.0007	5.99E-01
**6**	Non-supplemented	Big leaf maple	Control	Sg_0	0.0061	0.0034	0.0087	8.24E-06
**6**	Non-supplemented	Big leaf maple	Control	Sg_1	0.0015	-0.0011	0.0042	3.37E-01
**6**	Non-supplemented	Big leaf maple	Sg_0	Sg_1	-0.0045	-0.0065	-0.0025	1.02E-05
**6**	Non-supplemented	Sugar maple	Control	Sg_0	0.0034	-0.0001	0.0069	0.0581
**6**	Non-supplemented	Sugar maple	Control	Sg_1	0.0022	-0.0013	0.0057	0.277
**6**	Non-supplemented	Sugar maple	Sg_0	Sg_1	-0.0012	-0.0038	0.0015	0.525

Table S6. ANOVA test results for enzyme activity

**Enzyme**	**Comparison**	**DFn**	**DFd**	**F**	**p**
**Endocellulase**	Substrate	1	12	0.034	0.857
Endocellulase	Strain	2	12	0.026	0.975
Endocellulase	Substrate:Strain	2	12	1.133	0.354

Chitinase	Substrate	1	12	794.025	2.47E-12
Chitinase	Strain	2	12	237.198	2.26E-10
Chitinase	Substrate:Strain	2	12	251.71	1.59E-10

Table S7. Tukey's HSD test for ANOVA results for enzyme activity

Substrate	Condition 1	Condition 2	Estimate	Low confidence	High confidence	Adjusted p-value
**Substrate**	agar	maple	-9.36E-04	-0.001008	-0.000863	1.34E-12
**Strain**	0	10320	5.60E-04	0.0004515	0.0006685	2.86E-08
**Strain**	0	10321	8.74E-04	0.0007659	0.0009829	1.75E-10
**Strain**	10320	10321	3.14E-04	0.0002059	0.0004229	1.48E-05
**Substrate:Strain**	agar:0	maple:0	1.30E-05	-0.00018	0.0002062	1.00E+00
**Substrate:Strain**	agar:0	agar:10320	1.07E-03	0.0008798	0.0012662	4.26E-09
**Substrate:Strain**	agar:0	maple:10320	6.00E-05	-0.000133	0.0002532	8.94E-01
**Substrate:Strain**	agar:0	agar:10321	1.78E-03	0.0015913	0.0019777	5.14E-12
**Substrate:Strain**	agar:0	maple:10321	-2.27E-05	-0.000216	0.0001705	9.98E-01
**Substrate:Strain**	maple:0	agar:10320	1.06E-03	0.0008668	0.0012532	4.86E-09
**Substrate:Strain**	maple:0	maple:10320	4.70E-05	-0.000146	0.0002402	9.59E-01
**Substrate:Strain**	maple:0	agar:10321	1.77E-03	0.0015783	0.0019647	5.43E-12
**Substrate:Strain**	maple:0	maple:10321	-3.57E-05	-0.000229	0.0001575	9.87E-01
**Substrate:Strain**	agar:10320	maple:10320	-1.01E-03	-0.001206	-0.00082	7.87E-09
**Substrate:Strain**	agar:10320	agar:10321	7.12E-04	0.0005183	0.0009047	4.10E-07
**Substrate:Strain**	agar:10320	maple:10321	-1.10E-03	-0.001289	-0.000902	3.39E-09
**Substrate:Strain**	maple:10320	agar:10321	1.72E-03	0.0015313	0.0019177	6.77E-12
**Substrate:Strain**	maple:10320	maple:10321	-8.27E-05	-0.000276	0.0001105	7.06E-01
**Substrate:Strain**	agar:10321	maple:10321	-1.81E-03	-0.002	-0.001614	4.70E-12
